# Supplementary material for: Malnutrition in Infants Aged under 6 Months Attending Community Health Centres: A Cross Sectional Survey
Source: Nutrients. 2021 Jul 21;13(8):2489. doi: 10.3390/nu13082489 (PMC8398549; doi:10.3390/nu13082489)
Supplement: Supplementary file 1 [file nutrients-13-02489-s001.zip › nutrients-1276106-supplementary.pdf]

Supplementary Materials

Table S1. Characteristics of the 1,037 surveyed infants under six months of age, by study site.

	Jimma (n = 610)		Deder (n = 428)		Difference (Jimma - Deder)		
	mean or %	95% CI	mean or %	95% CI	difference	95% CI	p-value
Household members	5.3	5.1; 5.5	5.6	5.4; 5.9	-0.33	-0.62; -0.04	0.025
Household children aged <18 years	3.1	2.9; 3.2	3.4	3.2; 3.6	-0.35	-0.59; -0.11	0.005
Household head is male (%)	98.9	97.6; 99.5	99.1	97.5; 99.6	-0.21	-1.45; 1.04	0.745
Household head formal education							
No education (%)	36.0	32.1; 40.1	31.1	26.9; 35.7			
Grade 1-8 (%)	45.2	41.1; 49.4	45.8	41.1; 50.6			
Grade 9-12 (%)	10.7	8.40; 13.5	18.3	14.9; 22.3			
Technical and Vocational Education (%)	0.70	0.30; 1.90	0.50	0.10; 1.90			
College/University (%)	7.40	5.50; 9.90	4.30	2.70; 6.70			
Mother as main Caregiver (%)	98.9	97.6; 99.5	99.8	98.3; 100	-0.91	-1.87; 0.05	0.063
Grandparent help in infant care (%)	32.1	28.5; 35.9	41.1	36.5; 45.8	-9.00	-15.0; -3.03	0.003
Mother/Caregiver age (years)	25.7	25.2; 26.1	26.2	25.7; 26.7	-0.52	-1.20; 0.15	0.127
Mother/Caregiver is married (%)	98.7	97.4; 99.3	99.1	97.5; 99.6	-0.37	-1.66; 0.92	0.572
Mother/Caregiver age at marriage (years)	18.0	17.8; 18.2	16.6	16.4; 16.8	1.42	1.13; 1.70	<0.001
Time married (years)	7.66	7.22; 8.11	9.54	9.01; 10.1	-1.88	-2.57; -1.18	<0.001
Mother/Caregiver formal education							
No education (%)	38.5	34.7; 42.4	44.4	39.7; 49.1			
Grade 1-8 (%)	44.2	40.3; 48.2	48.1	43.4; 52.9			
Grade 9-12 (%)	11.5	9.16; 14.2	5.87	3.99; 8.54			
Technical and Vocational Education (%)	1.80	1.00; 3.22	0.47	0.12; 1.86			
College/University (%)	4.09	2.78; 5.99	1.17	0.49; 2.79			
Mother/Caregiver religion							
Muslim (%)	92.5	90.1; 94.3	96.7	94.5; 98.0			
Orthodox Christian (%)	5.60	4.00; 7.70	3.30	2.00; 5.50			
Protestant (%)	1.80	1.00; 3.20	0.00	0.00; 0.00			
Refused to answer (%)	0.20	0.00; 1.20	0.00	0.00; 0.00			

	Jimma (n = 610)		Deder (n = 428)		Difference (Jimma - Deder)		
	mean or %	95% CI	mean or %	95% CI	difference	95% CI	p-value
Infant's age (weeks)	12.4	11.9; 12.9	14.8	14.3; 15.4	-2.46	-3.20; -1.72	<0.001
Infant is male (%)	54.5	50.5; 58.4	56.6	51.8; 61.2	-2.07	-8.22; 4.08	0.509
Infant is singleton (%)	98.4	97.0; 99.1	98.8	97.2; 99.5	-0.46	-1.90; 0.97	0.527
Siblings aged <18 years	2.0	1.8; 2.1	2.4	2.2; 2.6	-0.47	-0.72; -0.22	<0.001
Infant's birth order							
1st (%)	27.7	24.3; 31.4	21.1	17.5; 25.3			
2nd (%)	22.8	19.6; 26.3	17.6	14.3; 21.5			
3rd (%)	14.9	12.3; 18.0	15.3	12.1; 19.0			
4th (%)	11.1	8.87; 13.9	13.9	10.9; 17.5			
5th (%)	10.3	8.13; 13.0	12.7	9.84; 16.2			
6th+ (%)	13.3	10.8; 16.2	19.5	16.0; 23.5			
Recent death of sibling (%)	11.6	9.30; 14.4	19.5	16.0; 23.5	-7.84	-12.39; -3.30	0.001
Infant ever breastfed (%)	98.4	97.0; 99.1	99.8	98.4; 100	-1.4	-2.51; -0.29	0.013
Infant received breastmilk as first food (%)	98.0	96.6; 98.9	93.2	90.4; 95.2	4.84	2.21; 7.48	<0.001
Infant was breastfed in the past 24 hrs (%)	94.6	92.5; 96.1	93.4	90.6; 95.4	1.17	-1.79; 4.13	0.438
Breastfeeding frequency in past 24 hrs (times)	10.5	10.2; 10.8	10.4	10.1; 10.6	0.11	-0.24; 0.47	0.534
Infant exclusively breastfed (%)	46.8	42.9; 50.8	51.9	47.1; 56.6	-5.07	-11.3; 1.12	0.108
Infant was bottle-fed (%)	13.4	10.9; 16.4	17.8	14.5; 21.8	-4.42	-8.96; 0.12	0.056
Infant fed any solid, semi-solid or soft foods (%)	1.64	0.88; 3.02	2.58	1.43; 4.60	-0.95	-2.76; 0.87	0.306
Bilateral pitting oedema (%)	1.15	0.55; 2.39	0.00	0.00; 0.00	1.15	0.30; 1.99	0.008
Weight (kg)	5.58	5.48; 5.68	5.68	5.57; 5.80	-0.1	-0.26; 0.05	0.185
Length (cm)	59.1	58.8; 59.5	60.4	59.9; 60.8	-1.24	-1.82; -0.67	<0.001
Mid-upper arm circumference (cm)	12.5	12.4; 12.6	12.4	12.3; 12.5	0.05	-0.10; 0.21	0.487
Triceps skinfold (mm)	7.9	7.7; 8.0	7.6	7.4; 7.7	0.27	0.06; 0.48	0.011
Subscapular skinfold (mm)	7.1	7.0; 7.3	6.3	6.1; 6.4	0.86	0.67; 1.04	<0.001
Head circumference (cm)	40.0	39.8; 40.2	40.8	40.6; 41.1	-0.79	-1.09; -0.49	<0.001
Knee-to-heel length (mm)	147	146; 148	149	148; 151	-2.39	-4.14; -0.64	0.007
Weight-for-age z-score (WAZ)	-0.47	-0.57; -0.37	-0.89	-1.01; -0.77	0.42	0.26; 0.58	<0.001

	Jimma (n = 610)		Deder (n = 428)		Difference (Jimma - Deder)		
	**mean or %**	**95% CI**	**mean or %**	**95% CI**	**difference**	**95% CI**	**p-value**
Length-for-age z-score (LAZ)	-0.28	-0.38; -0.18	-0.44	-0.58; -0.31	0.17	-0.01; 0.34	0.059
Weight-for-length z-score (WLZ)	-0.31	-0.40; -0.22	-0.7	-0.83; -0.58	0.39	0.23; 0.54	<0.001
Wasted (%)	7.45	5.61; 9.84	15.7	12.6; 19.5	-8.28	-12.3; -4.23	<0.001
Stunted (%)	7.54	5.69; 9.93	12.9	10.0; 16.4	-5.31	-9.11; -1.50	0.006
Underweight (%)	10.4	8.2; 13.1	16.4	13.2; 20.3	-6.00	-10.3; -1.72	0.006

Underweight, stunted and wasted was defined as WAZ, LAZ and WLZ <-2, respectively.

Table S2. Proportion of infants aged under 6 months with and without CIAF overlapping with low MUAC or underweight.

	0-5 weeks		6-10 weeks		11-15 weeks		16-20 weeks		21-25 weeks	
	n = 18		n = 57		n = 58		n = 59		n = 31	
**Infants with CIAF**	**%**	**95% CI**	**%**	**95% CI**	**%**	**95% CI**	**%**	**95% CI**	**%**	**95% CI**
MUAC <10.5cm	61.1	37.6; 80.4	17.5	9.68; 29.7	15.5	8.25; 27.3	10.2	4.62; 20.9	6.45	1.60; 22.6
MUAC <11.0cm	72.2	47.8; 88.1	42.1	30.0; 55.2	25.9	16.2; 38.6	18.6	10.6; 30.7	16.1	6.82; 33.6
MUAC <11.0cm if <17 weeks, <11.5 thereafter	72.2	47.8; 88.1	42.1	30.0; 55.2	25.9	16.2; 38.6	23.7	14.6; 36.2	32.3	18.2; 50.4
MUAC <11.0cm if <13 weeks, <11.5 thereafter	72.2	47.8; 88.1	42.1	30.0; 55.2	34.5	23.4; 47.6	23.7	14.6; 36.2	32.3	18.2; 50.4
MUAC <11.0cm if <7 weeks, <11.5 thereafter	72.2	47.8; 88.1	61.4	48.2; 73.1	39.7	27.9; 52.7	23.7	14.6; 36.2	32.3	18.2; 50.4
MUAC <11.0cm if <6 weeks, <11.5 thereafter	72.2	47.8; 88.1	61.4	48.2; 73.1	39.7	27.9; 52.7	23.7	14.6; 36.2	32.3	18.2; 50.4
MUAC <11.5cm	88.9	64.3; 97.3	61.4	48.2; 73.1	39.7	27.9; 52.7	23.7	14.6; 36.2	32.3	18.2; 50.4
Underweight	38.9	19.6; 62.4	64.9	51.7; 76.2	51.7	39.0; 64.3	67.8	54.9; 78.5	61.3	43.3; 76.7
**Infants without CIAF**	**n = 75**		**n = 210**		**n = 227**		**n = 184**		**n = 109**	
MUAC <10.5cm	40.0	29.5; 51.6	0.48	0.07; 3.33	0.00	0.00; 0.00	0.00	0.00; 0.00	0.00	0.00; 0.00
MUAC <11.0cm	64.0	52.4; 74.1	3.33	1.59; 6.85	0.00	0.00; 0.00	0.00	0.00; 0.00	0.00	0.00; 0.00
MUAC <11.0cm if <17 weeks, <11.5 thereafter	64.0	52.4; 74.1	3.33	1.59; 6.85	0.00	0.00; 0.00	0.00	0.00; 0.00	0.00	0.00; 0.00
MUAC <11.0cm if <13 weeks, <11.5 thereafter	64.0	52.4; 74.1	3.33	1.59; 6.85	0.88	0.22; 3.47	0.00	0.00; 0.00	0.00	0.00; 0.00
MUAC <11.0cm if <7 weeks, <11.5 thereafter	64.0	52.4; 74.1	9.52	6.21; 14.3	3.08	1.47; 6.34	0.00	0.00; 0.00	0.00	0.00; 0.00
MUAC <11.0cm if <6 weeks, <11.5 thereafter	64.0	52.4; 74.1	14.8	10.6; 20.3	3.08	1.47; 6.34	0.00	0.00; 0.00	0.00	0.00; 0.00
MUAC <11.5cm	80.0	69.3; 87.7	14.8	10.6; 20.3	3.08	1.47; 6.34	0.00	0.00; 0.00	0.00	0.00; 0.00
Underweight	--	--	--	--	--	--	--	--	--	--

CIAF: Composite Index of Anthropometric Failure. MUAC: Mid-Upper Arm Circumference.

Table S3. Proportion of CIAF categories overlapping with low MUAC.

	Wasted only n = 53		Wasted & Underweight n = 46		Wasted, Stunted & Underweight n = 13		Stunted & Underweight n = 51		Stunted only n = 37		Underweight only n = 23	
**Indicator**	**%**	**95% CI**	**%**	**95% CI**	**%**	**95% CI**	**%**	**95% CI**	**%**	**95% CI**	**%**	**95% CI**
MUAC <10.5cm	9.40	3.98; 20.8	17.4	8.94; 31.1	46.2	22.3; 71.9	31.4	20.2; 45.3	2.70	0.38; 16.9	8.70	2.18; 28.9
MUAC <11.0cm	11.3	5.17; 23.0	34.8	22.5; 49.5	69.2	40.9; 88.0	52.9	39.4; 66.1	8.11	2.63; 22.3	30.4	15.2; 51.6
MUAC <11.0cm if <17 weeks, <11.5 thereafter	11.3	5.17; 23.0	43.5	30.0; 58.0	92.3	60.9; 98.9	54.9	41.2; 67.9	8.11	2.63; 22.3	30.4	15.2; 51.6
MUAC <11.0cm if <13 weeks, <11.5 thereafter	11.3	5.17; 23.0	50.0	35.9; 64.1	92.3	60.9; 98.9	56.9	43.1; 69.7	8.11	2.63; 22.3	34.8	18.4; 55.7
MUAC <11.0cm if <7 weeks, <11.5 thereafter	20.8	11.9; 33.7	56.5	42.0; 70.0	92.3	60.9; 98.9	64.7	50.8; 76.5	8.11	2.63; 22.3	43.5	25.2; 63.7
MUAC <11.0cm if <6 weeks, <11.5 thereafter	20.8	11.9; 33.7	56.5	42.0; 70.0	92.3	60.9; 98.9	64.7	50.8; 76.5	8.11	2.63; 22.3	43.5	25.2; 63.7
MUAC <11.5cm	24.5	14.8; 37.8	58.7	44.1; 71.9	92.3	60.9; 98.9	64.7	50.8; 76.5	8.11	2.63; 22.3	43.5	25.2; 63.7

CIAF: Composite Index of Anthropometric Failure. MUAC: Mid-Upper Arm Circumference.
